# Death from Salivation in a Child

**Published:** 1840

**Authors:** 


### DEATH FROM SALIVATION IN A CHILD.

Our editorial colleague has received the details of a case of death from mercurial disease of the mouth, which occurred in the practice of one of his correspondents in the State of Missouri. Its publication entire could not extend our knowledge of the fatal character of the *gangrina mercurialis* of children, and we, therefore, notice it merely as as a warning—a renewed admonition against the excessive, and above all the *protracted* administration of calomel to children, before the time of puberty, and especially within the first climacterick. The patient, in this instance, was a girl of "delicate constitution," seven years old, affected with dysentery. The whole quantity of calomel given was about sixty grains; in small doses, some of which were administered *after* the patient's gums began to feel sore. This, considering the *age* of the patient, was, it must be admitted, in violation of the rule of safety. Nevertheless, the gentleman, who has thus had the courage and candor to report a sinister result of his own practice, did nothing more than what others are accustomed to do—generally, with impunity, but sometimes with fatal effect, or with what is scarcely less to be dreaded, permanent deformity. D.

